# Improving outcomes of surgery in advanced infiltrative thymic tumours: the benefits of multidisciplinary approach

**DOI:** 10.1177/03008916211023154

**Published:** 2021-06-22

**Authors:** Andrea Billè, Rajdeep Bilkhu, Giulia Benedetti, Gianluca Lucchese

**Affiliations:** 1 Department of Thoracic Surgery, Guy’s Hospital London, London, UK; 2 Department of Comprehensive Cancer Centre, School of Cancer and Pharmaceutical Sciences, King’s College London, Guy’s Hospital London, London, UK; 3 Department of Radiology, Guy’s Hospital London, London, UK; 4 Department of Cardiac Surgery, Guy’s Hospital London, London, UK

**Keywords:** Thymoma, resection, diaphragm, pericardium, reconstruction

## Abstract

**Background:**

For stage III or IVa thymic tumours, a multimodality approach is recommended. The role of surgery is to achieve complete resection.

**Aim:**

To present the outcomes of patients undergoing surgery for stage III or IVa thymoma.

**Methods:**

Retrospective review of patients undergoing open surgery for stage III or IVa thymoma between 2016 and 2020 at a single centre was performed. Preoperative imaging, treatment plan, surgical approach, and postoperative outcomes were analyzed.

**Results:**

Forty-seven patients underwent surgery for thymoma. Patients with clinical stage I/II thymoma or minimally invasive thymectomy were excluded. Thirteen patients with clinical stage III or IVa were included. Median sternotomy approach was used in four patients, of which one was redo sternotomy; a hemi-clamshell in four; and a combination of approaches in the remaining five patients. There was no postoperative mortality. Four patients had postoperative complications. Complete resection was achieved in all but two patients. At a median follow-up of 17.9 months, all patients were alive with no evidence of recurrence except one who died 4 months after surgery from coronavirus disease 2019 (COVID-19) pneumonia.

**Conclusions:**

Surgery for stage III and IVa thymoma is safe and can be achieved with complete macroscopic resection. To obtain adequate exposure of all structures involved in the tumour, combined surgical approaches can be used with no increased morbidity. The majority of patients, even after extrapleural pneumonectomy, did not receive adjuvant radiotherapy and had no evidence of local relapse.

## Introduction

Thymic tumors are rare and thymoma is the most common histology. Up to 40% of patients can present with locally advanced stage III or IVa thymoma.^1,2^ Surgery is the main treatment for early-stage thymic tumors. In patients with stage III or IV athymic tumors, a multimodality approach is recommended, with the aim of surgery being to achieve a complete macroscopic resection (R0),^1^ usually after neoadjuvant chemotherapy. Despite the lack of large series or randomised controlled trials, there is evidence that complete macroscopic resection in stage III and IVa thymomas can significantly increase disease-free and overall survival.^3–6^

Shapiro and Korst^1^ reviewed methods of surgical resection for patients with stage IVa thymic tumors and highlighted how the surgical approach is chosen based on disease extension and surgeon experience. Advanced stage thymomas may infiltrate the great vessels, pericardium, and pleura and extend into the lungs to varying degrees. In selected cases with great vessel involvement, a joint procedure with a cardiac surgeon is considered, particularly when aortic arch infiltration is present. In these cases, cardiopulmonary bypass might be indicated to perform extensive vascular resection and reconstruction. Sometimes a full pleurectomy, resection and reconstruction of the diaphragm, anatomical lung resection, or extrapleural pneumonectomy may also be required.

The choice of surgical access is fundamental to obtain access to the structures involved by the thymic tumor, in order to achieve a complete resection with clear margins. Our aim was to assess the outcomes of surgery in stage III and IVa thymoma and attempt to evaluate the best surgical approach and methods of reconstruction of involved structures in order to achieve a complete macroscopic resection, according to the extent of disease based on preoperative imaging and intraoperative findings.

## Methods

**Figure 1.**
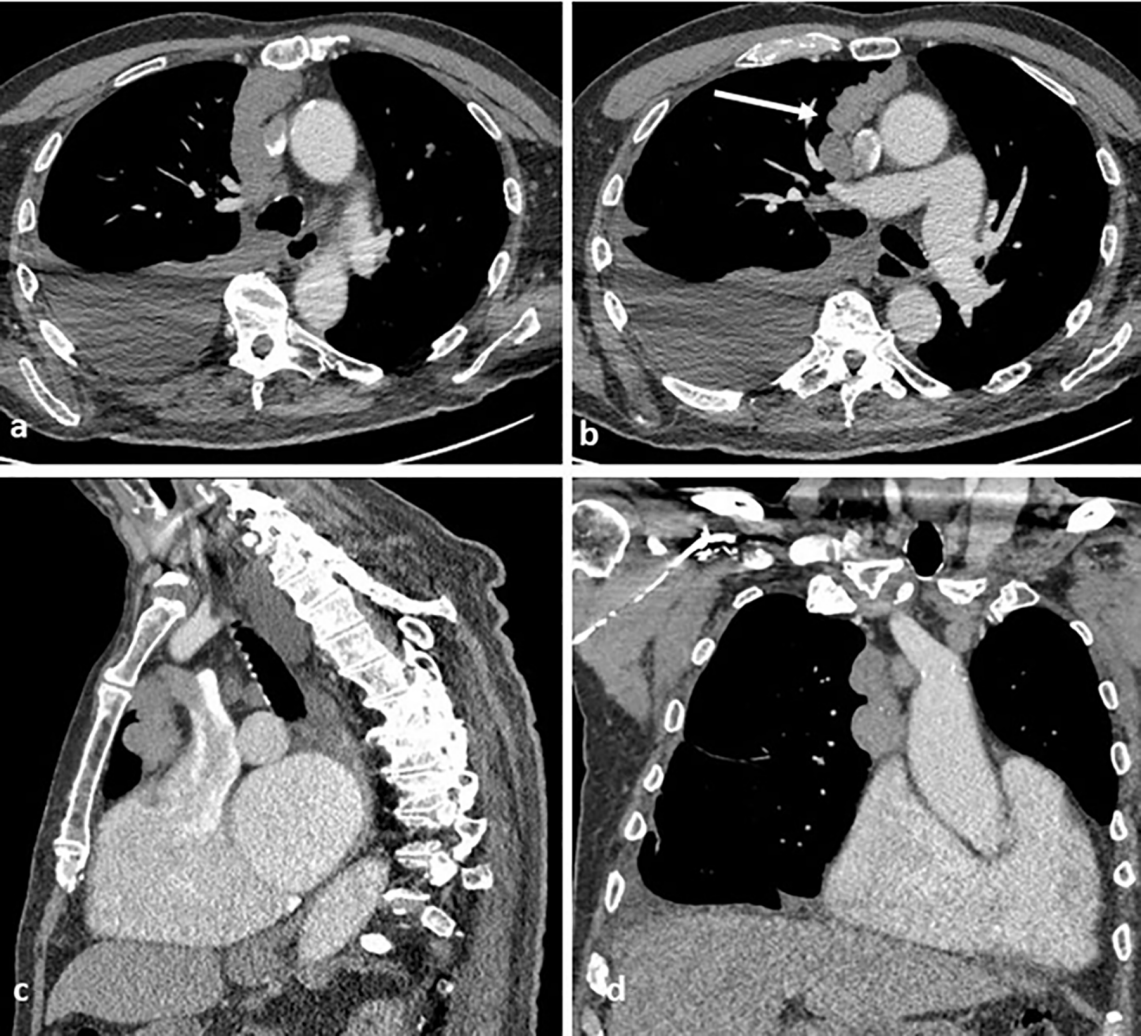
Images from preoperative computed tomography ([a] and [b]) show a right paramedian anterior mediastinal mass, with local involvement of the anterior aspect of the pericardium ([c] and [d]).

A retrospective review of patients presenting with advanced thymic tumours (stage III and IVa thymoma) treated surgically from 2016 to 2020 was performed. Patients with unresectable thymic carcinoma (n=5) were excluded. All patients consented to be included in the case series. Preoperative imaging, treatment plan, surgical approach, and postoperative outcome of patients were analysed and reviewed by the surgical team and the thoracic radiologist. All patients had a preoperative computed tomography (CT) scan (Figure 1) and a positron emission tomography (PET) scan. Magnetic resonance imaging (MRI) was performed when there was concern regarding invasion of the heart or great vessels. All patients had full lung function tests and echocardiogram as routine preoperative workup. For stage III disease, neoadjuvant chemotherapy was considered when there was concern regarding complete resection. In stage IVa disease, all patients underwent neoadjuvant chemotherapy. Adjuvant radiotherapy was considered for patients with incomplete resection or after stage IVa thymoma resection. All patients were followed up until March 2021.

Continuous data are reported as median and range, and categorical data are reported as count and percentage. Survival was measured from the date of surgery. Survival and prognostic factors were analysed by the Kaplan-Meier method.

The study conformed to the guidelines in the Declaration of Helsinki and was approved by the local ethics committee as a service evaluation project (Audit No. 5801).

## Results

Between 2016 and 2020, 47 patients underwent surgery for thymoma. Six cases were excluded from the analysis for repeated surgery due to disease recurrence (redo sternotomy for recurrent thymoma, n=2; thoracotomy for pleural recurrence, n=4). Patients who underwent robotic (n=9) and video-assisted thoracic surgery (n=2) thymectomy were also excluded. The remaining 30 cases underwent open thymectomy. Of these 30 patients, a total of 13 patients had clinical stage III or IVa thymoma and underwent open surgery and were included in the final analysis (Table 1). Eight (61.5%) patients were men; median age was 53 years (range 41–75). Three (23.1%) patients had myasthenia gravis at presentation; 1 (7.7%) patient had a performance status of 2. Two (15.4%) patients had stage III thymoma and underwent surgery without neoadjuvant chemotherapy considering the high probability of obtaining a complete resection. Six patients were diagnosed with stage IVa thymoma and underwent neoadjuvant chemotherapy followed by surgery, all of whom had only a partial response to chemotherapy.

**Table 1.** Characteristics of the cohort.

Characteristics	Values (total n = 13)
Age, y	53 (41–75)
Male sex	8 (61.5)
Myasthenia gravis	3 (23.1)
Clinical stage	
III	4 (30.8)
IVa	9 (69.2)
Induction treatment	5 (38.5)
Complications	
Haemodynamic instability	1 (7.7)
Respiratory failure	2 (15.4)
Prolonged air leak	1 (7.7)
Adjuvant treatment	1 (radiotherapy)
Recurrence at follow-up	0

Values expressed as median (range) or n (%).

Median sternotomy approach was used in 4 (30.8%) patients due to involvement of anterior mediastinum (Table 1); a hemi-clamshell (combination of partial median sternotomy and anterior thoracotomy) in 4 (30.8%); and a combination of approaches in the remaining 5 (38.4%) patients: median sternotomy and posterolateral thoracotomy (n=3) or hemi-clamshell and posterolateral thoracotomy (n=2) (Tables 2 and 3).

**Table 2.** Operative characteristics of patients undergoing thymectomy via median sternotomy only (n=4).

	Patient 1	Patient 2	Patient 3	Patient 4
Surgical approach	Median sternotomy	Redo median sternotomy	Median sternotomy	Median sternotomy
Lung resection	Wedge resection	No	Wedge resection	No
Pericardial resection	Yes	No	Yes	No
Diaphragmatic resection	No	No	No	No
Great vessel resection/reconstruction	Superior vena cava	No	No	No
Operative time, min	120	180	120	120
Estimated blood loss, mL	500	300	300	10
Transfusion (units packed red cells)	0	0	0	0
Chest drain removal, postoperative day	2	6	2	1
Length of hospital stay, d	7	12	4	4
Histology	Thymoma AB	Thymoma B1	Thymoma B2	Thymoma AB
Masaoka stage	III	III	III	III
Resection status	R0	R1	R0	R0

**Table 3.** Operative characteristics of patients undergoing thymectomy via combined surgical approaches (n=9).

	Patient 1	Patient 2	Patient 3	Patient 4	Patient 5	Patient 6	Patient 7	Patient 8	Patient 9
Side	Right	Right	Left	Left	Left	Left	Right	Right	Left
Surgical approach	hemi-clamshell	hemi-clamshell and posterolateral thoracotomy	hemi-clamshell	hemi-clamshell	median sternotomy and thoracotomy	hemi-clamshell	median sternotomy and thoracotomy	median sternotomy and thoracotomy	hemi-clamshell and posterolateral thoracotomy
Lung resection	Upper bilobectomy	Pneumonectomy	No	Pneumonectomy	Pneumonectomy	Lobectomy	Lobectomy	Extrapleural pneumonectomy	Lobectomy
Pericardial resection	No	Yes	Yes	Yes	Yes	Yes	Yes	Yes	Yes
Diaphragmatic resection	No	No	No	No	Yes	Yes	Yes	Yes	No
Great vessel resection and reconstruction	Yes	No	No	No	No	No	No	No	No
Operative time, min	400	600	180	270	420	540	400	600	240
Estimated blood loss, mL	1500	800	300	1475	800	1500	800	3700	1000
Transfusion (units packed red cells)	2	1	0	2	0	1	0	6	4
Chest drain removal, postoperative day	5	1	6	2	2	3	14	2	20
Length of hospital stay, d	6	6	9	49	6	52	48	29	8
Histology	Thymic carcinoma	Thymoma B2	Thymoma AB	Thymoma B2	Thymoma B2/B3	Thymoma B3	Thymoma B2	Thymoma A	Thymoma B3
Masaoka stage	III	IVa	I	IVa	IVa	IVa	IVa	IIIa	IVa
Resection status	R0	R0	R0	R0	R0	R1	R0	R0	R0

Ten (76.9%) patients had pericardial resection and repair with Gore-Tex (Gore Medical) mesh. In 4 cases, the pericardium was involved posteriorly and inferiorly to the inferior pulmonary veins; in these cases, the mesh was secured posteriorly through a posterolateral thoracotomy performed to complete the resection of the thymic tumour. In 2 cases, a great vessel resection was performed: in 1 case a partial superior vena cava resection and reconstruction with bovine pericardium patch was performed and in the other patient the innominate vein was resected but not reconstructed. In 2 cases, a partial diaphragmatic resection was performed and the diaphragm was directly repaired through the anterior approach. In 3 (23.1%) cases, an extensive diaphragmatic resection was performed and the diaphragm was repaired using a biological mesh (SurgiMend, Integra Lifesciences [n=2] and Permacol, Covidien [n=1]), positioned through a posterolateral thoracotomy.

**Figure 2.**
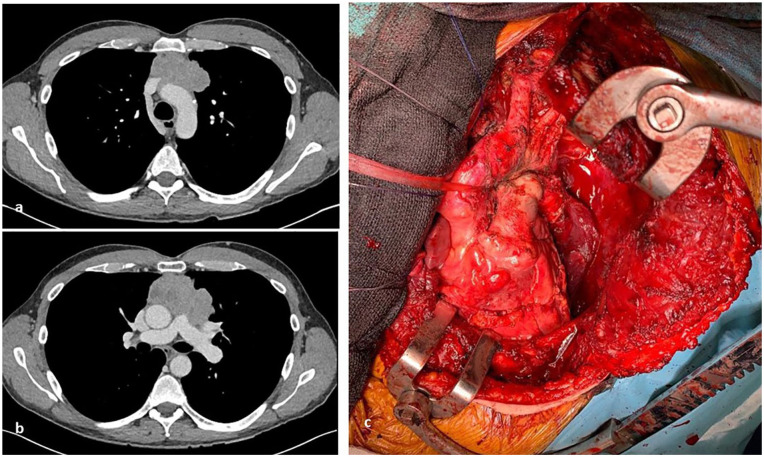
Preoperative computed tomography shows an anterior mediastinal mass with possible extension into the anterior aspect of great vessels (aortic arch, superior vena cava, left brachiocephalic vein, main pulmonary artery, proximal left pulmonary artery, left superior pulmonary vein) ([a] and [b]). Operative picture (c) demonstrates the great vessels following resection.

**Figure 3.**
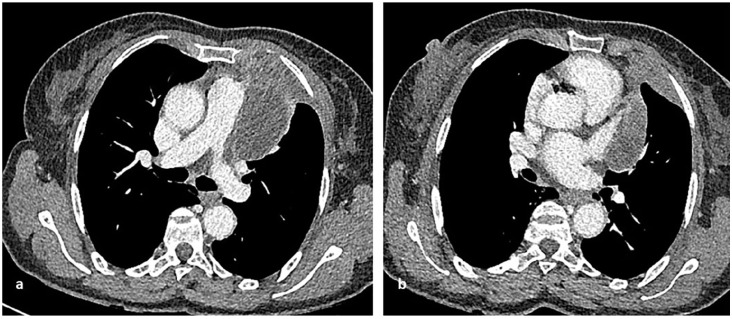
Preoperative computed tomography shows a left anterior mediastinal mass involving the main pulmonary artery, the proximal left pulmonary artery, and the left superior pulmonary vein but not the inferior. There is also involvement of the pericardium at the level of the left atrial appendage and of the left anterior para mediastinal thoracic wall. The mass does not extend inferiorly or posteriorly to the left hilum.

**Figure 4.**
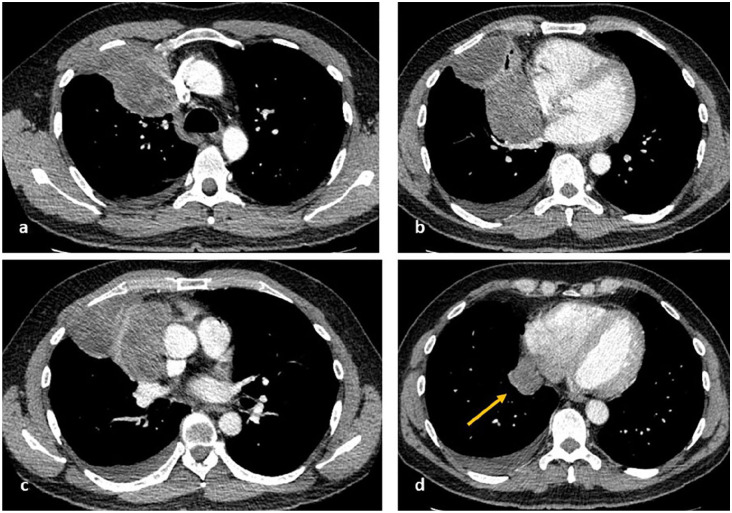
Preoperative computed tomography shows a right mediastinal mass with possible involvement of superior vena cava, right brachiocephalic vein, right main pulmonary artery, right superior and inferior pulmonary veins, and the pericardium surrounding the right atrium inferior to the right hila continuous with right basal para mediastinal pleural deposits (arrow). There is also extensive involvement of the right pleura with right anterior chest wall involvement.

Patients who underwent a hemi-clamshell approach had larger tumours (56 mm and 75 mm in maximum diameter), with possible involvement of the great vessels, including the left pulmonary artery in one case and the right pulmonary artery in the other case (Figures 2–4). These patients also had pericardial involvement, reaching but never extending behind the hilar below the lower pulmonary veins and never involving the posterior aspect of the pericardium. There was involvement of the chest wall in one case, but this was limited to the anterior paramediastinal chest wall adjacent to the primary tumour.

**Figure 5.**
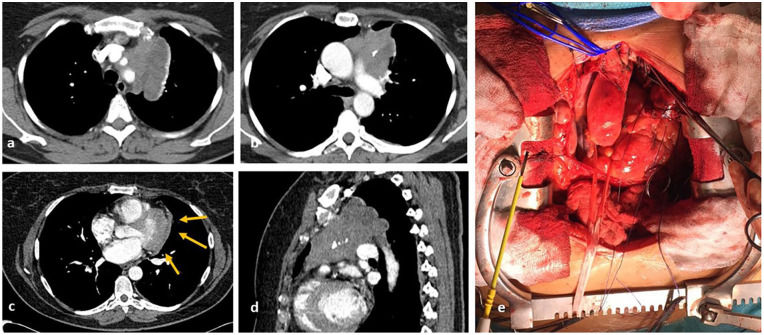
Preoperative computed tomography shows involvement of ascending aorta and main pulmonary artery, with possible extension surrounding the proximal descending aorta ([a], [b], and [d]). The mass also involves the pericardium around the left ventricle (arrows [c]). Complete macroscopic resection was achieved (d).

**Figure 6.**
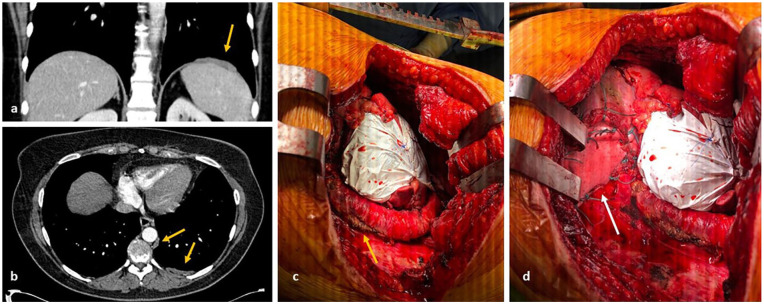
There are multiple pleural deposits within the left hemithorax with a left supradiaphragmatic deposit and a para-aortic deposit ([a] and [b]). View of the descending aorta and reconstruction of the diaphragm with Perma-Col (arrow) and pericardium with Gore-Tex ([c] and [d]) is shown.

Five patients who underwent combined approaches had large masses (>120 mm diameter) involving the great vessels, in one case extending posteriorly to surround the proximal descending aorta (Figures 4–6). Both cases had extensive pleural deposits within a single hemithorax and multiple pericardial deposits, located posterior to the hila or inferiorly to the pulmonary veins: one patient had a large pericardial deposit inferior and lateral to the left ventricular wall as well as left diaphragmatic deposits; the other had a deposit within the pericardium surrounding the posterior right atrium, continuous with posterior paramediastinal and paraspinal pleural deposits.

Four (30.8%) patients underwent pneumonectomy due to invasion of the hilum at the level of the pericardium and pleura. One of these four patients underwent extrapleural pneumonectomy due to the presence of extensive pleural disease. Four patients had a lobectomy/bilobectomy and 2 patients had a wedge resection. In one patient, the superior vena cava was resected and replaced without complication.

Intraoperative characteristics are summarized in Tables 1 and 2. There was no perioperative mortality. The median duration of chest drainage was 2 days (range 1–20 days); in patients who underwent only a sternotomy and an extra pneumonectomy, the drain was routinely removed within 48 hours. Median length of hospital stay was 8 days (range 4–49 days). Nine patients were electively admitted to intensive care. Postoperative complications were reported in 4 (30.8%) patients: cardiovascular instability in one, who required a posterolateral thoracotomy to reposition the pericardial mesh to prevent cardiac herniation; recurrent chest infection and tracheostomy to clear the secretions in another case; one patient required reintubation for respiratory failure; and one patient had prolonged air leakage. Only one patient received postoperative radiotherapy after extrapleural pneumonectomy. One patient who underwent left hemi-clamshell with R1 resection died on the 73rd postoperative day of coronavirus disease 2019 (COVID-19) pneumonia and did not receive radiotherapy.

One patient received postoperative radiotherapy after extrapleural pneumonectomy and developed severe heart failure; 12 (92%) patients did not have any adjuvant treatment and they did not show any evidence of local or distant recurrence. At a median follow-up of 20.9 months, all remaining patients were alive with no evidence of recurrence. Two patients had recurrent myasthenia gravis with no evidence of recurrent disease on CT or PET scan and they are on medical treatment. One patient died of COVID, but two had COVID during 6 month follow up and both recovered.

## Discussion

We have demonstrated that surgery can be performed in advanced infiltrative thymoma, with low perioperative mortality and with complete macroscopic resection in the majority of cases. Surgery may significantly improve the long-term outcome of patients with stage III and IVa thymoma.^7^ The goal is to achieve complete resection and this may require resection of the lung, the pericardium, the diaphragm, or the great vessels.

Our results are consistent with those of other groups. Kanzaki and colleagues^8^ reported on outcomes of patients with locally advanced thymoma who underwent surgery following chemotherapy or chemoradiotherapy over a 20-year period and reported complete resection being achieved in the majority of cases with no perioperative mortality. Similarly, Ried and colleagues^9^ assessed the outcomes of 22 patients with stage III or IVa thymoma over a 12-year period and reported no operative mortality, with complete macroscopic resection being achieved in 86% of patients.

In our series, only one patient had postoperative radiotherapy, developing significant toxicity; in the other patients, radiotherapy was not used as part of the multimodality approach, considering the possible cardiac toxicity. Our report shows that even in patients with pleural disease, when macroscopic resection is achieved radiotherapy may not be necessary to prevent risk of local relapse.

In order to achieve complete resection, as well as to prevent the incidence of perioperative complications, the choice of surgical approach is crucial to optimize tumour exposure, complete resection, and reconstruction. Median sternotomy is the approach of choice for early-stage large thymoma and in selected cases of locally advanced thymoma.^10^ Left manubriotomy may be considered provide a better exposure of the distal arch and left subclavian artery in case of involvement and vascular reconstruction.

In patients with locally advanced thymoma, a combination of surgical access can be used, according to the involvement of surrounding structures. Hemi-clamshell incision offers good surgical exposure for tumours reaching but not extending behind or below the pulmonary hila, with or without limited pleural involvement, but in the case of diffuse involvement of the posterior mediastinum or the diaphragm, it may be not sufficient.^11^ Posterolateral thoracotomy for tumours extending behind or below the pulmonary hila/extensive posterior pericardial involvement/multiple pleural involvement/diaphragmatic involvement may be required, as has been used in our series. This allows for pneumonectomy, extended pleurectomy, extrapleural pneumonectomy, and diaphragmatic resection to be performed with excellent exposure of the posterior mediastinum and diaphragm. The addition of posterolateral thoracotomy in the current series did not increase morbidity. Although not performed in the current series, a full clamshell incision may also be considered according to surgeon preference.

The availability of cardiopulmonary bypass (CPB) where there is extensive infiltration of the mediastinal vessels or in the event of brisk and uncontrollable bleeding from the major vessels has to be considered. The right atrium and vena cavae can be partially resected on CPB and reconstructed with glutaraldehyde-preserved autologous pericardium. Total resection of the superior and inferior vena cavae, main pulmonary artery, ascending aorta, aortic arch, and neck vessels and their reconstruction can be performed on CPB when needed. Although major aortic surgery or replacement of the great vessels would increase the risks of postoperative complications and mortality, it should be considered in young patients to achieve a complete macroscopic resection.

Reconstruction is an important component of surgery to prevent complications and improve quality of life after surgery. Reconstruction of the pericardium is crucial and represents the final part of the mediastinal component of the procedure. The pericardium is extensively resected, making sure to preserve at least one of the two phrenic nerves. In our series, it was replaced with a Gore-Tex membrane and fixed with a locking continuous 4-0 polypropylene suture to avoid purse-string effect and consequent constriction. The decision for a Gore-Tex membrane was determined by the low incidence of adhesions caused by the Gore-Tex in the long term, mainly with regards to the constrictive pericarditis. The correct position mesh is fundamental, mainly when lung resection is performed, to avoid heart herniation and cardiovascular instability, as in one of our patients. This patient underwent an extrapleural pneumonectomy through a left hemi-clamshell with pericardial resection; the mesh was originally positioned via the hemi-clamshell. The difficulties in reaching the posterior pericardial edges resulted in postoperative cardiac herniation and cardiovascular instability. The patient required a thoracotomy to reposition the mesh posteriorly. Planning a combined midline and thoracotomy approach in those with expected posterior pericardial involvement allows for the pericardial mesh to be placed securely to prevent cardiac constriction or reduced venous return.

Patients with pleural involvement may present with droplet metastases involving the parietal and diaphragmatic pleura. In these cases, resection of the diaphragm may be necessary. In cases of limited diaphragmatic resection, we recommend a direct repair, which can be performed through a median sternotomy or a hemi-clamshell. In those requiring more extensive diaphragmatic resection, a posterolateral thoracotomy may be necessary to reach the posterior diaphragmatic recess and remove the diaphragm posteriorly. In these patients, we recommend use of a Gore-Tex or biological mesh to reconstruct the defect. In our series, we did not experience any incomplete resection or postoperative herniation using a combined approach in case of extensive diaphragmatic involvement.

Our study is limited by lack of follow-up data, but outcomes of surgery for advanced thymoma have been reported by some groups, with a disease-free survival of 50% at 10 years with overall survival of 87% at 10 years follow-up.^8^ Another group recently reported outcomes of 54 patients over a 7-year period who underwent surgery for thymoma stage III and IVa with overall and disease-free survival of 77.8% and 75.9% at 5 years.^12^ As such, surgery should continue to be advocated in these patients. We showed that this surgery can be performed safely despite the COVID-19 pandemic but the risk of acquiring COVID-19 pneumonia is high and the risk of mortality is higher.

## Conclusion

We have demonstrated that surgery for stage III and IVa thymoma is feasible and safe and complete macroscopic resection can be achieved in the majority of cases. The surgical approach should be tailored to ensure exposure of all the structures involved by the tumour and to achieve complete resection. A dedicated multidisciplinary team and surgical synergy is key to success in these challenging cases. Given the limited number of patients referred with advanced tumours, referral to experienced, high-volume centres is recommended to ensure that patients with advanced disease receive surgical resection to improve prognosis.
